# Drug Resistance among Pulmonary Tuberculosis Patients in Calabar, Nigeria

**DOI:** 10.1155/2013/235190

**Published:** 2013-08-26

**Authors:** Akaninyene Otu, Victor Umoh, Abdulrazak Habib, Soter Ameh, Lovett Lawson, Victor Ansa

**Affiliations:** ^1^Department of Internal Medicine, University of Calabar Teaching Hospital, PMB 1278, Cross River State, Nigeria; ^2^Department of Medicine, Aminu Kano Teaching Hospital, PMB 3452, Kano, Nigeria; ^3^Department of Community Medicine, University of Calabar Teaching Hospital, PMB 1278, Cross River State, Nigeria; ^4^Zankli Medical Centre, Plot 1021 Shehu YarAdua Way, Abuja, Nigeria

## Abstract

*Background*. This study aimed to determine the pattern of drug susceptibility to first-line drugs among pulmonary TB patients in two hospitals in Calabar, Nigeria. *Methods*. This was a descriptive cross-sectional study carried out between February 2011 and April 2012. Sputum samples from consecutive TB patients in Calabar were subjected to culture on Lowenstein-Jensen (LJ) slopes followed by drug susceptibility testing (DST). The DST was performed on LJ medium by the proportion method. *Results*. Forty-two of the 100 *Mycobacterium tuberculosis* strains were found to be resistant to at least one drug. Resistance to only one drug (monoresistance) was found in 17 patients. No strains with monoresistance to rifampicin were found. Resistance to two drugs was found in 22 patients, while one patient was resistant to both three and four drugs. MDR TB was seen in 4% (4/100). The independent variables of HIV serology and sex were not significantly associated with resistance (*P* > 0.05). *Conclusion*. There was a high prevalence of anti-TB drug resistance in Calabar.

## 1. Introduction

Tuberculosis (TB), an ancient infectious disease caused by *Mycobacterium tuberculosis*, is the leading cause of death due to an infectious agent globally. It is both preventable and treatable [1, 2]. The World Health Organization (WHO) records an average of nine million new TB cases annually and about 5000 TB deaths daily [1]. TB and human immunodeficiency virus (HIV) coinfection and the exponential increase in drug resistance are greatly responsible for the resurgence of TB [3]. Other identified factors include neglect of TB control by governments, poor management of programmes, poverty, population growth, and rapid uncontrolled urbanization [4]. 

Drug-resistant TB is a case of TB (usually pulmonary) excreting bacilli resistant to one or more anti-TB drugs [5]. Acquired drug resistance results from exposure to a single drug due to irregular drug supply, inappropriate prescription, or poor adherence to treatment. This suppresses the growth of bacilli susceptible to that drug while permitting multiplication of drug-resistant organisms. Primary or initial drug resistance occurs when such drug resistant bacilli are transmitted to other people [5]. 

Resistance to one anti-TB drugs is known as mono resistance. Poly resistance is resistant to two or more anti-TB drugs, but not to both isonazid & rifampicin. Multidrug-resistant TB (MDR TB) is resistant to at least isoniazid and rifampicin, the two key first-line anti-TB drugs in short course chemotherapy [5]. These forms of TB do not respond to the standard six-month treatment with anti-TB drugs [6].

Drug resistance poses a major challenge to the control of TB. It requires a longer duration of therapy with close monitoring and specialized treatment facilities. Patients remain infectious for longer periods and drug-resistant TB causes accelerated disease leading to substantial mortality. The average treatment duration is longer than that of drug susceptible TB. This makes adherence to treatment more challenging to achieve. Treatment of drug-resistant TB employs second-line medications which are less effective and display cross-resistance and high toxicity profiles. Injectable drugs are also utilized for long durations with the attendant side effects. Also, these second-line medications are very expensive and not readily available at primary care level.

*Mycobacterium tuberculosis* divides slowly both in vitro and in vivo and is inherently resistant to many conventional antibiotics. For successful treatment of TB, the antibiotics should penetrate macrophages and caseous material and be effective against dormant bacilli which could be reactivated later on [7]. Rifampicin, isoniazid, and pyrazinamide are bactericidal agents which have good sterilizing activity. Ethambutol and streptomycin are less effective than other first-line agents [8]. Resistance to first-line anti-TB drugs has been linked to mutations in at least 10 genes: *katG*, *inhA*, *ahpC*, *kasA*, and *ndh *for isoniazid resistance; *rpoB *for rifampicin resistance; *embB *for ethambutol resistance; *pncA *for pyrazinamide resistance; *rpsL *and *rrs *for streptomycin resistance [9].

Nigeria has the tenth largest burden of TB cases in the world [10]. The 2010 WHO estimates for Nigeria put the prevalence of TB at about 320000 (199 per 100000) cases with an incidence of 210000 (133 per 100000) and a mortality of 33000 (21 per 100000). The case detection rate (CDR) was low at 40%, while the treatment success rate (TSR) among new smear-positive cases for 2009 was 83% [10]. The 2011 WHO report states that Nigeria has an estimated MDR-TB rate of 2.2% and 9.4% among new and retreatment TB cases, respectively. Nigeria is therefore ranked the 15th among the 27 high burden countries for MDR-TB [10].

TB drug resistance is not a new occurrence in Nigeria. It was described as early as 1976 by Fawcett in Zaria [11]. Since then, there have been other reports on TB drug resistance in various parts of the country. Kehinde and colleagues at University College Hospital Ibadan, Nigeria, in 2005, tested 56 culture positive specimens of *Mycobacterium tuberculosis* for sensitivity to first-line anti-TB drugs. Thirty (53.6%) of the culture-positive isolates were resistant to both isoniazid and rifampicin, while 26 (46.4%) were susceptible [12]. Lawson and colleagues in 2011 reported DST for streptomycin, isoniazid, rifampicin, and ethambutol performed on 428 culture-positive samples on BACTEC-MGIT 960. Eight percent of the specimens cultured were MDR TB with varying levels of resistance to individual and multiple first-line drugs. HIV status was documented in 71%. There was no association between MDR-TB and HIV coinfection (*P* = 0.9) and gender (*P* > 0.2) [13]. A study by Idigbe et al. among 48 HIV seropositive and 50 HIV seronegative prison inmates in Lagos, Nigeria, who had TB showed similar results of significant difference in initial TB drug-resistance rates between the seropositive and seronegative inmates [14]. 

Despite these researches, there are no studies reflecting the prevalence of TB drug resistance in the southeastern part of Nigeria. TB culture and drug-resistance testing are not routinely carried out as part of the laboratory workup of persons with TB in Nigeria as sputum microscopy for acid fast bacilli is the mainstay. This present study aimed to determine the prevalence and pattern of drug resistance to first-line anti-TB drugs among newly diagnosed pulmonary TB patients in health institutions in Calabar, Nigeria. 

## 2. Materials and Methods

### 2.1. Study Area

The study took place in two health facilities in Calabar: the University of Calabar Teaching Hospital (UCTH) and the Lawrence Henshaw Hospital (LHH). Calabar is the state capital of Cross River State. Cross River State is one of the states in the southeastern part of Nigeria. According to the national census figures, Cross River State has a total population of 2,888,966 with 1,492,465 males and 1,396,501 females [15]. 

### 2.2. Study Participants

Participants were consecutive patients seen in TB clinics or admitted into the medical wards of UCTH and the LHH with a diagnosis of pulmonary TB but who were yet to start anti-TB chemotherapy. The diagnosis of pulmonary TB was made using Ziehl-Neelsen (ZN) staining of sputum to identify acid-fast bacilli (AFB). A positive test was considered to be the identification of at least 10–99 AFB per 100 oil immersion fields. 

### 2.3. Ethical Considerations

This research received ethical approval from the Health Research and Ethics Committee of the Cross River State Ministry of Health, Nigeria. High ethical standards were maintained throughout the entire duration of the study. A signed informed consent was obtained from each participant after detailed explanation of the study objectives to them. The participants were free to withdraw from the study at any point they felt like.

### 2.4. Inclusion Criteria

Participants included in the study were those who gave consent and were ≥15 yrs of age with a diagnosis of pulmonary TB as confirmed by at least two out of three sputum samples positive for AFB. 

### 2.5. Exclusion Criteria

Pulmonary TB patients who were currently receiving anti-TB chemotherapy, patients with extra pulmonary TB, and those who could not expectorate were also excluded.

### 2.6. Procedure

Three sputum samples were collected from each participant. Either two on-the-spot samples with one early morning specimen (from out-patients) or three early morning samples (from admitted patients) were obtained. Sputum samples were analysed for AFB by ZN method. Only intending participants with at least two sputum samples positive for AFB were admitted into the study. This conformed with the WHO's standard definition of a smear-positive TB case which stipulates at least two initial sputum smear examinations (direct smear microscopy) positive for acid-fast bacilli [16, 17]. 

The sputum samples were then transported from Calabar to Zankli Medical Centre, Abuja in cold boxes. Zankli is widely recognised reference centre for the isolation of *Mycobacterium tuberculosis *in Nigeria [18]. At Zankli, the samples were mixed with equal volume of 4% sodium hydroxide for digestion and decontamination. Digested specimens were washed in sterile distilled water by centrifugation at 3000 g for 15 minutes. The centrifugation process was repeated for bacilli sedimentation, and 0.1-0.2 mL of homogenate was inoculated on paired Lowenstein-Jensen (LJ) egg-based slopes. All the cultures were incubated at 37°C with weekly examination for growth of *Mycobacterium *spp. until eight weeks. All isolates showing positive reaction for catalase and nitrate reductase assays were subcultured on LJ slopes to obtain pure and confluent growth required for drug susceptibility testing (DST). A total of 120 patients were recruited with two ZN positive samples. Only one sputum sample was cultured and analysed for drug susceptibility per participant. However, 100 isolates were eventually identified as *Mycobacterium tuberculosis* following culture on LJ slopes. Twenty samples were no longer viable as they did not yield any growth following culture on LJ slopes.

DST for these 100 pulmonary isolates using isoniazid, rifampicin, ethambutol, and streptomycin was performed on LJ medium by the proportion method. The LJ media were prepared for each of the four drugs, with final concentrations of 0.2 *μ*g isoniazid, 2 *μ*g ethambutol, 40 *μ*g rifampicin, and 4 *μ*g streptomycin. Bacterial suspensions were inoculated by concentrations (S1–S4) into drug-free and drug-containing slopes. Susceptibility or resistance was recorded when the proportion of bacteria in drug-containing medium to that of drug-free medium is <1 or ≥1, respectively.

Data generated from the study was entered and analysed using the Statistical Package for Social Sciences (SPSS) IBM version 19.0. 

## 3. Results

The mean age of the final study population was 34 ± 11. There were 53 males and 47 females and they were predominantly Christians (99%). Most (96%) of the participants had received some form of education, and, majority (52%) were from the Efik tribe. Trading was the commonest occupation among the participants as shown in Table 1. Fifty of the 100 participants were HIV seropositive.

**Figure 1:**
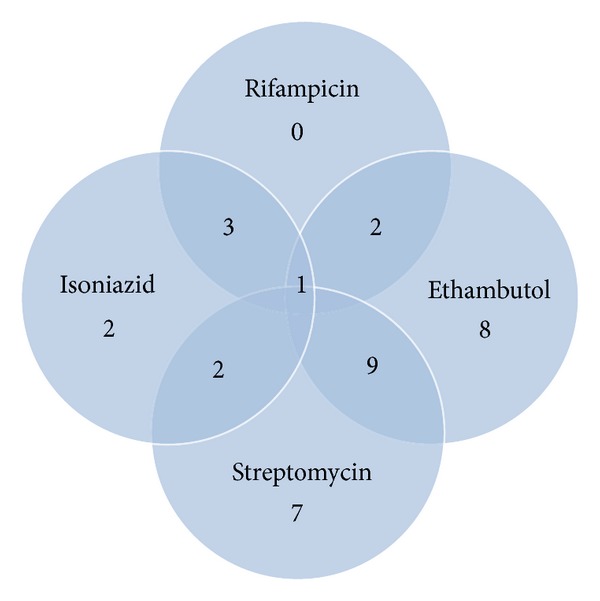
Resistance to one or more drugs in cultures of all TB subjects.

**Figure 2:**
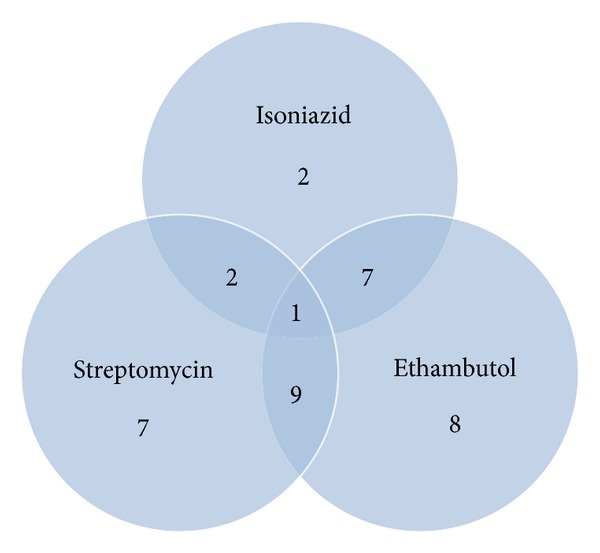
Resistance to isoniazid, ethambutol, and streptomycin.

Forty-two of the 100 patients evaluated were found to be resistant to at least one drug (42%; 95% CI: 32.3–51.7%). Figures 1 and 2 show the patterns of resistance to one or more anti-TB drugs. Resistance to only one drug (monoresistance) was found in 17 patients and was as follows: isoniazid (H): 2% (2/100); ethambutol (E): 8% (8/100); streptomycin (S): 7% (7/100). No strain with monoresistance to rifampicin was found. With respect to resistance to two drugs, 3% (3/100) were resistant to R + H; 1% (1/100) was resistant to R + E; 2% (2/100) were resistant to H + S; 7% (7/100) were resistant to H + E; 9% (9/100) were resistant to S + E. Resistance to three drugs H+E+S was seen in 1% (1/100) and 1% (1/100) was resistant to all four drugs. MDR-TB was seen in 4% (4/100).

The drug with the highest resistance profile was ethambutol occurring in 28/42 (66.6%). This was followed by streptomycin resistance in 20/42 (47.6%) and isoniazid resistance in 16/42 (38%) of the resistant cases. Resistance to rifampicin was recorded in 14/42 (33.3%) of the resistant cases.

The independent variables were then cross-tabulated against the dependent variable (resistance to anti-TB drugs) to identify statistically significant associations. The independent variables tested were HIV serology and sex. These two variables were not significantly associated with resistance (*P* > 0.05) as shown in Table 2.

## 4. Discussion

This study found drug resistance in 42% of the pulmonary TB patients tested in Calabar, Southeastern Nigeria. Monoresistance rates in this present study were high for ethambutol (8%) and streptomycin (7%), but rifampicin monoresistance was not demonstrated. 

Drug-resistant TB ultimately develops from the inadequate treatment of active pulmonary TB. This may result from poor prescribing practices among medical doctors with poor drug selection and insufficient treatment duration [19]. Systemic problems, through inadequate public health resources and unpredictable drug supplies, also play a role [20]. Erratic or selective compliance to treatment and default among clients is another key factor as it causes *Mycobacterium tuberculosis* to be exposed to sublethal doses for insufficient durations. This could thus result in treatment failure and foster emergence of drug-resistant TB and may increase the cost of treatment [21].

This low level of resistance to rifampicin in the present study is in keeping with findings of Idigbe et al. who reported a 2% resistance to rifampicin in contrast to 38% resistance to isoniazid seen in Lagos, Nigeria [17]. The high susceptibility of the *Mycobacterium tuberculosis* strains to rifampicin may be linked to its high bactericidal activity. Thus, the isolates showed a higher resistance rates to the less effective ethambutol and streptomycin. The low frequency of resistance to rifampicin is also generally ascribed to the new history of use of rifampicin, especially in African countries. 

The high prevalence of anti-TB drug resistance in this study was mirrored by Idigbe and colleagues who reported that 56% of the TB patients studied in Lagos, Nigeria, were resistant to at least one anti-TB drug tested [22]. Similarly, Lawson et al. found lower monoresistance rates of 31% in Abuja, Nigeria, using automated BACTEC liquid culture media [18]. In Jos, Nigeria, Ani and colleagues described a monoresistance prevalence of 15% and multidrug resistance of 31% among follow-up TB patients [23]. All these studies highlight the growing threat posed by drug-resistant TB in Nigeria.

In other parts of Africa, the situation does not appear to be very different. Desta in Ethiopia found even higher rates of TB drug resistance of 58.7% [24]. Another study in Ethiopia also reported high resistance rates of 21.4% to at least one anti TB drug [25]. Kabedi et al. recorded a very high rate of primary drug resistance in a cross-sectional study in Kinshasa, Congo [26]. The primary resistance of *Mycobacterium tuberculosis* to first-line drugs among the subjects studied was 43.5%.

Outside of Africa, drug-resistance rates have been similarly high. A report of National Baseline Survey of drug-resistant TB (2007-2008) in China showed that resistance to at least one anti-TB drug among new cases with smear positive pulmonary TB was 35.2% [27]. Borann in Cambodia also reported high resistance to at least one anti-TB drug of 52% [28]. Dam in India also reported a high resistance to first-line anti-TB drugs (isoniazid, rifampicin, streptomycin, and ethambutol) of 39.2%. This was demonstrated among treatment failure pulmonary TB patients [29]. 

The high TB drug resistance rates in Calabar, Nigeria, thus reflect the rising trend in other parts of the country and indeed the world at large. Identification of the magnitude of the problem in Nigeria still remains a challenge as there is inadequate laboratory capacity to perform diagnostic testing among TB patients. Thus, the estimated numbers of drug-resistant TB cases in Nigeria have been based on mathematical modelling rather than empirical studies. Only few centres in Nigeria offer automated BACTEC TB culture and DST. The six zonal reference TB culture laboratories are yet to be fully operational [18]. This falls short of the WHO recommendation of one TB culture facility per five million population [30]. These shortcomings in the Nigerian system have militated against the effective control of drug resistant TB in Nigeria.

Several biological mechanisms linking drug-resistant TB to HIV infection have been suggested [31]. Drug malabsorption in HIV-infected patients, especially rifampicin and ethambutol, can lead to drug resistance and has been shown to lead to treatment failure. Drug-resistant strains may be less virulent and preferentially lead to disease progression in immunocompromised patients, as opposed to immunocompetent individuals. Data supporting this hypothesis has not yet been observed in humans [32]. However, the univariate analysis in the present study did not reveal an association between HIV infection and anti-TB drug resistance. This is similar to reports from earlier studies in Nigeria. Lawson and colleagues in their study of 32 TB culture-positive patients in Abuja reported no association between HIV infection and anti-TB drug resistance [18]. In a subsequent bigger study by Lawson and colleagues, they again failed to find an association between MDR-TB and HIV coinfection (*P* = 0.9) in Nigerian patients [12]. Pereira et al. studied a total of 70 *M. tuberculosis *isolates, 30 from HIV seropositive, and 40 from HIV seronegative TB patients in India. They found that the prevalence of drug-resistant *M. tuberculosis *isolates among HIV seropositive TB patients was similar to that of HIV seronegative TB patients indicating that HIV infection may not be associated with drug-resistant TB [33]. 

Three studies in South Africa also found no association between HIV infection and MDR-TB. In a retrospective study in Durban, 2.4% of 42 HIV coinfected and 11.5% of 253 HIV-negative patients had MDR-TB [34]. A prospective study of hospitalized TB patients in Cape Town found an MDR-TB prevalence of 3.2% in 93 HIV coinfected patients, compared to 2.6% in 115 HIV-negative patients [35]. In gold miners, the MDR-TB rate was 5.3% among 207 HIV coinfected and 6.5% among 215 HIV-negative miners [36]. From the foregoing, it appears that studies from very high TB burden countries like Nigeria, India, and South Africa have consistently reported no association between HIV and TB drug resistance.

This present study was limited by the fact that sputum AFB positivity was relied upon for admission. Thus, the TB/HIV coinfected persons who were AFB negative were excluded. Availability of rapid diagnostic tools such as GeneXpert MTB/RIF assays could have circumvented this. Also, the present study was limited to health institutions in Calabar. Larger countrywide drug-resistance surveys will provide a better estimate of the burden of TB drug resistance in Nigeria.

## 5. Conclusion

The high rates of TB drug resistance in the present study need to be urgently addressed. Laboratory facilities for rapid TB culture and DST are needed across Nigeria for early and accurate diagnosis of drug-resistant cases. This remains an important step in managing TB drug resistance in Nigeria.

## Figures and Tables

**Table 1:** Demographics of subjects.

Characteristics	Number *n* = 100
Age (years)	
Mean (SD)	34 (11.1)
Sex	
Male	53
Female	47
Religion	
Christian	99
Muslim	1
Educational status	
None	4
Primary	25
Secondary	52
Tertiary	19
Tribe	
Efik	52
Ekoi	7
Ibibio	27
Annang	5
Ibo	8
Others	1
Occupation	
Civil servant	8
Banker	1
Student	16
Manual worker	22
Petty trader	27
Retired	1
Unemployed	9
Others	16

**Table 2:** Demographic, clinical characteristics of TB participants according to general resistance to anti-TB drugs.

Variable	Number of resistant participants	Crude OR (95%)	*P* value
Sex			
Male	23	0.6 (0.3–1.3)	0.3
Female	19		
HIV serology			
Positive	22	1.2 (0.5–2.6)	0.9
Negative	20		
